# A Marine Organism Detection Framework Based on the Joint Optimization of Image Enhancement and Object Detection

**DOI:** 10.3390/s21217205

**Published:** 2021-10-29

**Authors:** Xueting Zhang, Xiaohai Fang, Mian Pan, Luhua Yuan, Yaxin Zhang, Mengyi Yuan, Shuaishuai Lv, Haibin Yu

**Affiliations:** 1 School of Mechanical Engineering, Hangzhou Dianzi University, Hangzhou 310018, China; zxt@hdu.edu.cn; 2 Ocean Technology and Equipment Research Center, Hangzhou Dianzi University, Hangzhou 310018, China; 3 School of Electronics and Information, Hangzhou Dianzi University, Hangzhou 310018, China; ygrybz1314@hdu.edu.cn (X.F.); ai@hdu.edu.cn (M.P.); bruce_zyx@hdu.edu.cn (Y.Z.); claire_my@hdu.edu.cn (M.Y.); lvshuai@hdu.edu.cn (S.L.); 4 Ocean College, Zhejiang University, Zhoushan 316021, China; 5 Shandong Radio Monitoring Station, Jinan 250013, China; yuanluhua@126.com

**Keywords:** marine organism detection, marine monitoring, underwater image enhancement, underwater object detection, joint optimization, generative adversarial mechanism

## Abstract

Underwater vision-based detection plays an increasingly important role in underwater security, ocean exploration and other fields. Due to the absorption and scattering effects of water on light, as well as the movement of the carrier, underwater images generally have problems such as noise pollution, color cast and motion blur, which seriously affect the performance of underwater vision-based detection. To address these problems, this study proposes an end-to-end marine organism detection framework that can jointly optimize the image enhancement and object detection. The framework uses a two-stage detection network with dynamic intersection over union (IoU) threshold as the backbone and adds an underwater image enhancement module (UIEM) composed of denoising, color correction and deblurring sub-modules to greatly improve the framework’s ability to deal with severely degraded underwater images. Meanwhile, a self-built dataset is introduced to pre-train the UIEM, so that the training of the entire framework can be performed end-to-end. The experimental results show that compared with the existing end-to-end models applied to marine organism detection, the detection precision of the proposed framework can improve by at least 6%, and the detection speed has not been significantly reduced, so that it can complete the high-precision real-time detection of marine organisms.

## 1. Introduction

The rapid development of underwater observation technology provides underwater optical vision with very broad application prospects. As a typical application of underwater optical vision, underwater visual target detection plays an increasingly important role in underwater security [1,2,3,4], marine exploration [5,6], fish farming [7] and marine ecology [8,9]. Therefore, the achievement of underwater autonomous operation through visual target detection completion by use of underwater optical images has become a research hotspot in the field of computer vision [1].

Given the absorption and scattering effects of water on light, underwater images [10,11,12,13,14,15,16,17,18] are characterized by low color contrast, bluish-green tones [12,13,14,15], noise pollution [16,17], and therefore poor quality relative to ordinary images [19,20]. In addition, the carrier is usually moving in actual underwater operation, and the collected images will inevitably include motion blur which, in turn, results in a lack of clear contour structure and rich texture information in underwater images [21]. Thus, the underwater background environment will affect the accuracy of underwater visual target detection and limit its practical engineering applications.

The current underwater visual target detection methods can be roughly divided into two categories according to the underwater environment containing the visual imaging equipment: underwater target detection on the basis of the ideal hypothesis [18,22,23,24,25,26,27,28] and on a complex environment [29,30,31,32,33]. The underwater target detection method according to the ideal assumption is suitable for the ideal underwater environment with good lighting conditions and relatively static carrier and can complete simple engineering applications. Those methods include traditional target detection and general target detection approaches based on deep neural networks. For the traditional target detection technique, corresponding features on the basis of the human analysis of specific underwater tasks are firstly selected, such as color [22], texture [23], and geometric features [18]. Subsequently, models based on the features are developed so as to achieve underwater target detection tasks. However, these features depend on human experience and task characteristics to a large extent, thereby generating poor environmental adaptability. Traditional detection models are also relatively limited. To further improve the target detection performance of the model, deep neural networks are introduced into underwater target detection. The more popular methods include SSD [26], YOLO [27], and Faster RCNN [28]. Compared with the traditional method in which modeling and detecting are performed after extracting features, the general target detection technique based on the deep neural network can automatically complete feature extraction (which improves environmental adaptability) and also greatly improves the detection performance. Nevertheless, these methods overlook the actual underwater operating environment. Once the actual environment deviates from the ideal assumption, the detection performance of these methods will be considerably reduced. 

Underwater target detection methods on the basis of a complex environment initiate a typical two-step strategy specific to the defects of underwater imaging. First, the low color contrast, bluish-green tone [12,13,14,15], and noise pollution [16,17] in the picture are addressed through a series of image enhancement preprocessing steps, such as max-RGB [29], shades of gray [30], and brightness mapping [31]. Afterwards, the detection and classification of marine organisms are completed with the help of a detection model based on the deep neural network. This approach enhances the model’s adaptability to complex underwater environments. Nonetheless, inconsistencies in the image preprocessing and the optimization goals of target detection models exist in the staged method, and both features cannot be jointly tuned. Furthermore, the combination of multiple models is not conducive to project implementation.

To solve the above problems, some researchers embed an image preprocessing algorithm into the detection network framework [29,32,33] and complete the algorithm through the neural network so that it can be unified in the deep learning framework and complete the end-to-end training and recognition. For example, Huang et al. first embedded image enhancement into a VGG16 feature extraction network on the basis of an extended data set and completed the detection and recognition of underwater organisms in the URPC data set in the extracted feature maps with the use of the Faster R-CNN network [33]. Some scholars also proposed a novel sample weighted loss function invert multi-class Adaboost to reduce the impact of noise on the detection network [29]. Fan et al. introduced a composite connection backbone to enhance feature representation to address the blur and texture distortion in underwater data sets [32]. The main problems in the existing end-to-end approach are as follows: (1) Only certain image quality deterioration factors are considered in the image enhancement algorithm. As the enhancement work is only partially completed, more complex underwater operating environments cannot be explored. (2) As the marine organisms in the underwater images usually vary in scales and size and are uneven in terms of position distribution because of certain limitations of the underwater movement of the camera’s carrier, the multi-scale problem of target recognition is inevitable. However, multi-scale problems are not specially processed in the feature extraction network in the existing framework. (3) The network model used is relatively old. A one-way multi-layer convolutional neural network is only employed for feature extraction, and this approach is shallow and cannot consider both global and local feature information.

An end-to-end underwater target detection model is thus proposed to specifically address the problem of marine organism detection in complex environments. Compared with the existing staged model and end-to-end model, the target feature extraction network structure is improved, able to consider multiple factors that lead to the degradation of underwater imaging quality and can achieve high-precision marine organisms under the premise of satisfying real-time detection. The advantages are as follows:

(1) An end-to-end underwater object detection framework is proposed, which can jointly optimize the enhancement module and the detection module so as to improve the problem of large information loss in the existing two-stage model with first enhancement and then detection. In the enhancement module, the introduction of three sub-modules as denoising, color correction, and deblurring can alleviate the effects of the three main factors that lead to a significant drop in underwater imaging quality at the same time.
(2) The feature pyramid network is introduced given the problem of difficult detection caused by the uneven distribution of sizes and positions of different types of marine organisms in underwater images. High-quality feature extraction of marine organisms can be achieved by the use of the combination of deep semantic information and shallow detail information at different levels.
(3) Dynamic label allocation, dynamic smoothing of L1 loss, and dynamical adjustment of the IoU threshold are introduced because of the difficulty of generating enough positive samples from the network arising from the clustering effect of marine organisms. Thus, the contribution of positive samples in the training model is increased and model training is accelerated.

## 2. Detection Framework

### 2.1. Overall Structure

**Figure 1:**
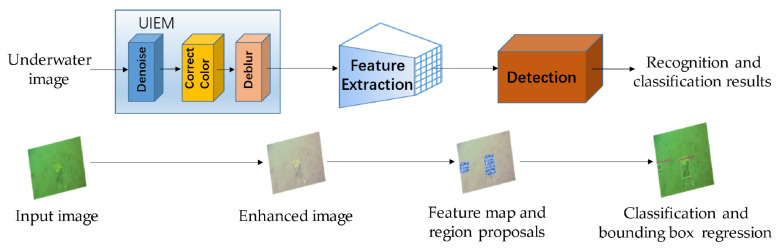
The overall structure of the proposed framework.

An end-to-end marine organism detection framework suitable for complex underwater environments is proposed in this work. The overall structure of the framework is shown in Figure 1. The framework consists of the underwater image enhancement module (UIEM), the feature extraction module, and the back-end detection module.

In the image enhancement module, three sub-modules are adopted to complete the step-by-step denoising, color correction and deblurring of underwater images. In the feature extraction module, multiple residual networks (ResNets) are employed to build a feature pyramid to complete the high-level feature extraction of the enhanced underwater image. Meanwhile, in the feature extraction process, a top-down path conveys high-level strong semantic features and a bottom-up path supplements the low-level strong positioning information. The two feature transmission methods are combined to better cope with the problems of different scales and uneven location distribution of marine organisms. In the detection module, a two-stage detection network is adopted to complete target detection. To enable the prediction box in the two-stage network to be better matched for the marine organisms with different scales, an adaptive threshold method is applied to dynamically adjust the classifier and regression on the basis of the sample distribution. Furthermore, the pre-training of the module is completed by utilizing the self-built data set for the most complex UIEM in the framework. Therefore, the training of the entire framework can be completed end-to-end so that the enhancement and the detection modules can be jointly tuned.

### 2.2. Underwater Image Enhancement Module

As mentioned, noise, color cast, and motion blur are the main factors for the deterioration of underwater image quality. Therefore, the image enhancement module proposed in this work is focuses on these three factors, including the corresponding three sub-modules for denoising, color correction, and deblurring. To enable joint tuning of the enhancement module and subsequent detection modules, all three sub-modules of the enhancement module must be pre-trained. The fact that the original image and the enhanced image cannot be obtained in pairs in the actual acquisition process of underwater images should be taken into consideration, that is, no paired data can be used for training. Correspondingly, with the practice in [29] as a reference, underwater images with high imaging quality are selected in this research from the existing data set, and various influencing factors are manually added to obtain paired underwater images that can be used for pre-training of the three sub-modules.

#### 2.2.1. Denoising Sub-Module

**Figure 2:**
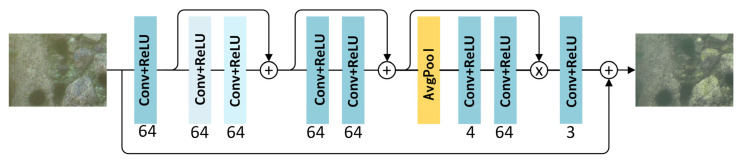
The diagram of the blind denoising sub-module network.

A one-stage blind denoising sub-module based on the feature attention mechanism is adopted to denoise and enhance the noisy underwater images. The detailed network structure of the blind denoising sub-module is shown in Figure 2. The network consists of eight convolutional layers. The kernel size of each convolutional layer is 3 × 3, the stripe is 1, the two light blue convolutional layers are hollow convolutions and their dilations are 2 and 4, respectively. The number below each layer represents the number of output channels. Each convolutional layer is followed by a rectified linear unit (ReLU). First, one convolution and two hollow convolutions are used to extract features from the input. The hollow convolution can enlarge the receptive field so that each convolution output contains a larger range of information. Then, a residual block containing two convolutions is employed to learn the features. Finally, a residual network that utilizes convolution to generate feature attention is adopted to reconstruct the image with an aim to explicitly model the interdependence between feature channels. The residual network on the basis of feature attention can automatically identify the importance of each feature channel through learning. This degree of importance serves as a reference to improve useful features and suppress features that are not very useful for the current task so as to achieve the removal of the underwater complex noise.

#### 2.2.2. Color Correction Sub-Module

**Figure 3:**
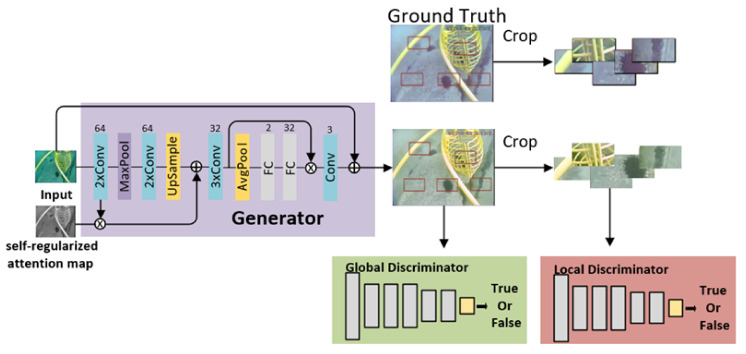
Overall structure of the color correction sub-module based on generative adversarial networks.

In the color correction sub-module, the enhancement of the underwater color cast image is completed with the help of a generative adversarial mechanism. The overall structure is shown in Figure 3. The CNN network oriented by self-attention is adopted as a generator, and a self-regularized perceptual loss technique is introduced to constrain the feature distance between the color cast input image and the enhanced image. There are eight 3 × 3 convolutional layers followed by leaky ReLU and batch normalization in the generator part, so as to make the data distribution consistent and avoid the disappearance of gradient. The pool layer adopts max pooling with a kernel size of 2. In Figure 3, the number above each layer represents the number of output channels. Moreover, a dual discriminator structure is included to balance global and local image color correction. Note that the convolutional layers of the entire discriminator network are 4 × 4 small convolution kernels with a step size of 1, and they are followed by leaky ReLU to obtain more features.

In the generator part, the normalized brightness channel value I of the input RGB image is taken, and then the 1-I point-by-point difference is employed as our self-regularized feature map and is multiplied with the feature map to constrain the generated image content. An attention mechanism is also added to the generative network. The channels are weighted through full connection so as to emphasize effective information and suppress invalid information. Meanwhile, the generator combined with the jump connection can better inhibit the channels that will increase the distance of the enhanced image feature.

In the discriminator part, adversarial loss is used to minimize the difference in light distribution between the target image and the image output by the generator. As the pixel-level discriminators usually do not show better performances on spatially changing images, when some local areas of the input image need to be enhanced differently from other parts, the global image discriminator alone usually cannot provide the required adaptability. For example, in our actual project, the underwater lighting is only provided by artificial light sources, and the illumination around the lighting point is sufficient. However, a place far away from the lighting point is darker, the absorption of light at different distances is also inconsistent, and the degree of color cast also varies. To improve the adaptability of the local area, a local discriminator is introduced in the discriminator of the color correction network on the basis of the global discriminator. The local discriminator obtains the input by randomly cropping local color patches from the generator output and the real image and learns to distinguish whether they are real (from a well-performing image) or fake (from the output of the generator). Conversely, the global discriminator evaluates the probability that the real image is more real than the generated image and then guides the generator to restore a more credible fake image.

#### 2.2.3. Deblurring Sub-Module

**Figure 4:**
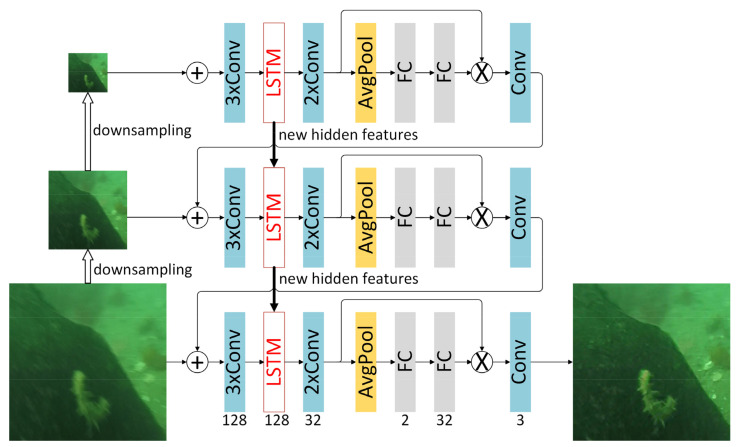
The diagram of the multiscale-based deblurring network.

Underwater images are blurred because the scene is changing during the camera exposure. For example, motion blur will occur if the object is captured during the movement of the underwater robot equipped with an underwater camera [21]. As for the reasons for blurring, by drawing on a network structure that has been successfully applied in image deblurring, a RNN network that gradually restores clear images with different resolutions in the feature extraction layer of the pyramid structure is used in the deblurring sub-module to achieve a blind image deblur. The network structure is shown as in Figure 4. The network consists of three subnetworks. Each layer has the same structure and is composed of six convolutional layers, two fully connected layers and one long short-term memory (LSTM). Different layers correspond to different size inputs. The kernel size of each convolutional layer is 5 and the stripe is 1. The “coarse to fine” scheme is adopted in this network, that is, clear images are gradually recovered at different resolutions in the pyramid. Moreover, the encoder–decoder network is employed to combine the output of the network layers at different scales with the help of a top-down path. Accordingly, the output of the low-resolution top layer will stack and input into the encoder along the channel with the high-resolution input, and a CNN network is utilized to gradually transform the input image into a feature map with a smaller size but more channels. In the network, the connection of the encoder output into a LSTM network can enable the feature map to obtain the hidden features of low-resolution input, and then deliver the new hidden features to the LSTM in the higher-resolution subnetwork. The network also enables the model to pay greater attention to features with substantial information when restoring images by the use of the attention mechanism, thereby allowing the network to make better use of global information.

### 2.3. Feature Extraction Network

Network depth is essential for many visual recognition tasks. However, deeper neural networks are more difficult to train. With the increase of the network depth, higher training errors will occur in the training process, and the accuracy will reach saturation and drop rapidly [34]. To address this problem, multiple residual blocks are used to construct a feature pyramid so as to extract features from the input. These residual blocks can optimize the network more easily and can improve accuracy after a significant increase in depth. Given that certain information loss will be generated by the previous image enhancement module and some information helpful for detection will be lost after enhancement, feature extraction can be performed by entering multiple residual modules after adding the input and the enhanced output according to the adaptive weight overlap through a long-term skip connection.

**Figure 5:**
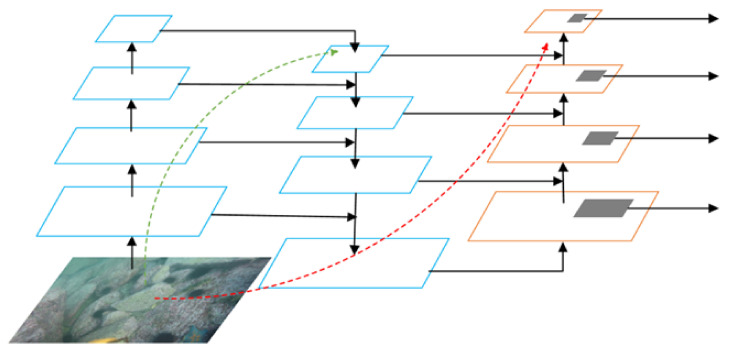
Residual feature extraction network with a combination of top-down and bottom-up paths.

The overall structure of the feature extraction network is shown in Figure 5. Low-level features contain less semantic information, but the target location is accurate. Conversely, high-level features contain richer semantic information, but the target location is relatively rough [35,36]. The marine organisms to be detected and recognized by the proposed framework are mainly small creatures such as sea urchins, sea cucumbers, and scallops whose individual sizes in the acquired images are very small. Thus, semantic information and target location are both crucial in the detection process. Therefore, referring to the method in [37], we use a combination of two feature transfer methods to pay attention to the semantic information and target location information simultaneously. Specifically, the top-down path is first adopted to transfer the strong semantic features in the high level, and then a bottom-up path is added to supplement the feature map so that the strong positioning features of the low-level are transferred up.

### 2.4. Detecting Networks

A two-stage detection network integrates feature extraction, proposal extraction, bounding box regression, and classification. In the network, the region proposal network (RPN) is directly used to generate detection frames, and the extracted features are shared in the RPN layer and subsequent network layers to determine target categories and position predictions. This approach greatly improves the overall network performance. Given its better overall performance, the two-stage network is selected as the detection network in this study. In the two-stage detection network, the selection of intersection over union (IoU) threshold is vital to the performance of the classifier. If the IoU threshold is set low, guaranteeing the sample quality would be difficult. By contrast, if the IoU threshold is set too high, an imbalance between positive and negative samples will occur, and a higher IoU threshold will easily lead to the loss of the small-scale target frame that the proposed framework emphasizes. To obtain a reasonable IoU threshold, especially for discriminating IoU thresholds that are too high, the IoU thresholds of positive/negative samples are gradually adjusted according to the proposal distribution in the training process. Meanwhile, the β in the smooth L1 loss is adjusted through the regression label distribution so as to change the regression loss function, with an aim to adapt to the distribution change of the regression label and ensure the contribution of high-quality samples to the training.

## 3. Training Processes

As discussed in Section 2.2, all three sub-modules of the UIEM must be pre-trained to enable the enhancement module and subsequent detection modules to be jointly tuned. Therefore, the training process of the framework proposed in this work will be divided into two parts: the pre-training of the UIEM and the end-to-end training of the target detection module. Note that the paired data sets used for sub-modules pre-training are all obtained by manually adding various influencing factors to high-quality images.

### 3.1. Pre-Training of the UIEM

#### 3.1.1. Pre-Training of the Denoising Sub-Module

The data set used for the pre-training of the denoising sub-module is formed by adding Gaussian, speckle, and salt-pepper noises to the selected high-quality underwater images. During the pre-training of the denoising sub-module, L1 loss is used to represent the deviation between the output image of the network and the noise-free image, and the loss function that must be minimized is shown as in Equation (1):

(1) $${Loss}_{Denoise}=\frac{1}{N}\sum_{i=1}^{N}{\left\|y\left(x_{i}\right)-y_{i}^{*}\right\|}_{1}$$

where $x_{i}$ is the input image with noise; $y\left(x_{i}\right)$ is the output of the blind denoising network; $y_{i}^{*}$ is the true value corresponding to $x_{i}$, that is, the noise-free image; and N is the number of pairs of noisy images and corresponding noiseless images in a batch during training.

#### 3.1.2. Pre-Training of the Color Correct Sub-Module

The data set used for the pre-training of the color correct sub-module is formed by the artificial color correction of the original underwater image. Considering that if the commonly used cross entropy is employed as the loss, the generator will no longer optimize those generated images recognized by the discriminator as real images, even if these generated images are still far from the decision boundary of the discriminator. Therefore, the least squares GAN loss (LSGAN) is used as the loss function of the color cast correction sub-module, which is defined as follows:

(2) $$\begin{array}{c} L_{D}^{Global}=E_{x_{r}∼P_{real}}\left[{\left(D\left(x_{r},x_{f}\right)-1\right)}^{2}\right]+E_{x_{f}∼P_{fake}}\left[D{\left(x_{f},x_{r}\right)}^{2}\right]\\ L_{G}^{Global}=E_{x_{f}∼P_{fake}}\left[{\left(D\left(x_{f},x_{r}\right)-1\right)}^{2}\right]+E_{x_{r}∼P_{real}}\left[D{\left(x_{r},x_{f}\right)}^{2}\right] \end{array}$$

(3) $$\begin{array}{c} L_{D}^{Local}=E_{x_{r}∼P_{real}}\left[{\left(D\left(x_{r}\right)-1\right)}^{2}\right]+E_{x_{f}∼P_{fake}}\left[{\left(D\left(x_{f}\right)-0\right)}^{2}\right]\\ L_{G}^{Local}=E_{x_{r}∼P_{fake}}\left[{\left(D\left(x_{f}\right)-1\right)}^{2}\right] \end{array}.$$

$L^{Global}$ represents the loss of the global discriminator, $L^{Local}$ represents the loss of the local discriminator, *D* represents the discriminator network, and $x_{r}$ and $x_{f}$ are the sampled values from the real distribution and the pseudo distribution, respectively. The local area in the local discriminator is obtained by randomly cropping five color blocks of the same size from the output image and the real image each time.

To reduce the instability of the generator network and improve performance, a self-regularized perceptual loss is added to the loss function by learning from the practice of Johnson et al. in [38]. The detailed method involves the input of the generated image into the trained feature extraction network (the VGG network is used in this article) to obtain the feature map and the subsequent comparison with the output of the real image input into the feature extraction network in the same layer. The difference between the fake image created by the generator and the input image is measured by the feature space distance as

(4) $$L_{SRP}\left(I^{L}\right)=\frac{1}{W_{i}H_{i}}\sum_{x=1}^{W_{i}}\sum_{y=1}^{H_{i}}{\left(\phi_{i}\left(I^{L}\right)-\phi_{i}\left(G\left(I^{L}\right)\right)\right)}^{2}$$

where $I^{L}$ represents the color cast input, $G\left(I^{L}\right)$ represents the enhanced output, and $\phi_{i}$ represents the feature map obtained from the feature extraction network. *i* represents its *i*th pooled feature map. $W_{i}$, $H_{i}$ is the dimension of the extracted feature map.

Combining Equations (2)–(4), the total loss function of the color correct sub-module is:

(5) $${Loss}_{Color}=L_{SRP}+L_{G}^{Global}+L_{G}^{Local}$$

**Figure 6:**
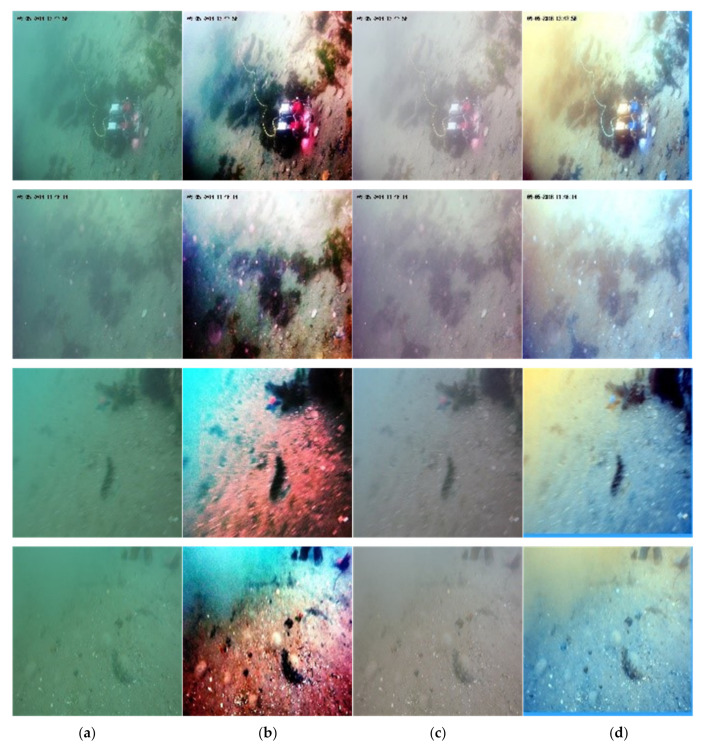
Color cast correction results of different methods: (**a**) original image, (**b**) histogram equalization, (**c**) ground Truth and (**d**) proposed module.

The pre-training result of the color correct sub-module is shown in Figure 6, where the ground truth image is obtained by the artificial color correction of the original color cast underwater image.

#### 3.1.3. Pre-Training of the Deblurring Sub-Module

For the pre-training data set of the deblurring sub-module, underwater images with motion blur are generated by averaging the continuous short exposure frames in the video taken by the high-speed camera with reference to the method in [39,40]. The resulting images are vivid as they can simulate the complex camera shake and object movement common in real photos. During deblurring network training, L2 norm loss is used to measure the difference between the network output image and the real image as

(6) $$Loss_{Deblur}=\sum_{i=1}^{n}\frac{1}{N_{i}}{\left\|y^{i}-y_{*}^{i}\right\|}_{2}^{2}$$

In the formula, $y^{i}$ and $y_{*}^{i}$ respectively represent the picture after blind deblurring and the real picture; $N_{i}$ represents the size of a batch.

### 3.2. Detection Network

The discussion in Section 2.4 indicates that training with different IoU thresholds will result in classifiers with different qualities. To achieve high-quality object detection, the classifier with the highest possible IoU threshold is needed for classifier training. Considering the dynamic characteristics of training, that is, the distribution of the proposal will be changed over time, the method we adopt is to automatically update the IoU threshold according to the distribution of the proposal. As the training progresses, the threshold for distinguishing the positive and negative samples will be gradually increased, which reflects the improvement of the quality of the classifier. Specifically, the average value of the K-th largest IoU of multiple iterations is taken as our threshold. Initially, a low threshold is adopted as the RPN is incapable of generating enough high-quality proposals. As the training proceeds, the RPN prediction quality is improved, enough high-quality proposals are gradually obtained, and the threshold at this time will automatically become higher. Therefore, a RPN with very high quality under high IoU will be obtained.

Multi-task objective loss function is adopted in the RPN network training. The network can simultaneously complete the training of the classification and regression tasks. To adapt to the changes in distribution and compensate for high-quality samples, dynamic SmoothL1 (DSL) is adopted as our position regression loss function to gradually focus on high-quality samples. DSL will dynamically adjust the position regression loss on the basis of the statistical data of the regression label to reflect the accuracy of positioning:

(7) $$L\left(p_{i},p_{i}^{*}\right)=\frac{1}{N_{cls}}\sum L_{cls}\left(p_{i},p_{i}^{*}\right)+\lambda \frac{1}{N_{reg}}\sum p_{i}L_{reg}\left(t_{i},t_{i}^{*}\right)$$

(8) $$L_{cls}\left(p_{i},p_{i}^{*}\right)=-\log[p_{i}p_{i}^{*}+\left(1-p_{i}\right)\left(1-p_{i}^{*}\right)]$$

(9) $$L_{reg}\left(t_{i},t_{i}^{*}\right)=DSL\left(t_{i},t_{i}^{*},\beta_{now}\right)=\left\{\begin{array}{c} \frac{0.5{\left|t_{i}-t_{i}^{*}\right|}^{2}}{\beta_{now}},if\;\left|t_{i}-t_{i}^{*}\right|<\beta_{now},\\ \left|t_{i}-t_{i}^{*}\right|-0.5\beta_{now},\;otherwise. \end{array}\right.$$

$L_{cls}$ is the target classification loss, and $L_{reg}$ is the position regression loss. $p_{i}$ is the probability that the current *i*-th anchor may be an object after the network judgement, and $p_{i}^{*}$ is the probability that the *i*-th anchor is marked as an object. $t_{i}$ is the offset parameter of the *i*-th private anchor relative to the region proposal. $t_{i}^{*}$ is the offset parameter of the *i*-th private anchor relative to the ground truth, and λ is the coefficient that weighs the classification loss and the regression loss. $N_{cls}$ is the network batch size, $N_{reg}$ is the number of Anchor positions, and $\beta_{now}$ is the dynamically adjusted hyperparameter.

The candidate area obtained through the RPN network involves the input to the RoI pooling layer so as to obtain a fixed-length feature vector. Finally, feature vector is input to the fully connected layer to obtain the category and location of the candidate area.

## 4. Experiment Details

### 4.1. Evaluation Indicator

Recall, precision, average precision (AP), mean average precision (mAP), and mAP@ [0.5:0.05:0.95] are the currently popular evaluation indicators for target detection performance. All these indicators can be calculated with the help of a confusion matrix. Their definitions are as follows:

- Recall:

(10) $$Recall=\frac{TP}{TP+FN}$$

In Equation (10), TP and FN are respectively the number of positive classes predicted as positive classes in the confusion matrix and the actual number of negative classes predicted as negative classes in the confusion matrix:

- Precision:

(11) $$precision=\frac{TP}{TP+FP}$$

In Equation (11), TP and FP correspond to the number of positive classes predicted as positive classes in the confusion matrix and the number of negative classes predicted as positive classes in the confusion matrix.

- Average precision (AP): With 0.05 as the interval, the average of all the accuracies of a certain category with the IoU threshold value from 0.5 to 0.95.
- Mean Average Precision (mAP): The average value of APs in all detection categories under a certain IoU threshold.
- mAP@ [0.5:0.05:0.95]: represents the average mAP at different IoU thresholds (from 0.5 to 0.95 in steps of 0.05).

### 4.2. Data Sets

Two data sets are involved in this work: the pre-training data set of each sub-module in the UIEM and the marine organism data set used for framework performance testing. As obtaining paired data sets is difficult in actual engineering, paired training sets are acquired for pre-training through the manual addition of noise, color correction, and generation of motion blur.

The marine organism data set used for the overall framework performance test is provided by the “Underwater Robot Picking Competition” organized by the Natural Science Foundation of China [28]. Some of the images were obtained by underwater robots with cameras. The robots are remote control robots designed for the fishing of marine organism. The data set contains four different marine organisms with cultivation value, such as sea urchin, sea cucumber, starfish, and scallop. The training set includes 4200 randomly selected images, and the validation set has 800 images. The hyperparameters of the model are finely adjusted through the validation set so that the fit of the training set is improved. Afterwards, 1200 underwater images that participated in the training as a test set are used to evaluate the generalization ability of the overall framework.

## 5. Experimental Results

In verifying the overall performance of the proposed framework, detection precision is ascertained on the underwater data set with the help of the test set, and the performance of the proposed framework is compared with the target detection network that has been widely used on land or underwater. In addition, in order to verify the role of the three sub-modules used for image enhancement in the proposed framework, we also conduct ablation experiments on these three sub-modules.

### 5.1. Experimental Results Obtained with the Underwater Data Set

**Figure 7:**
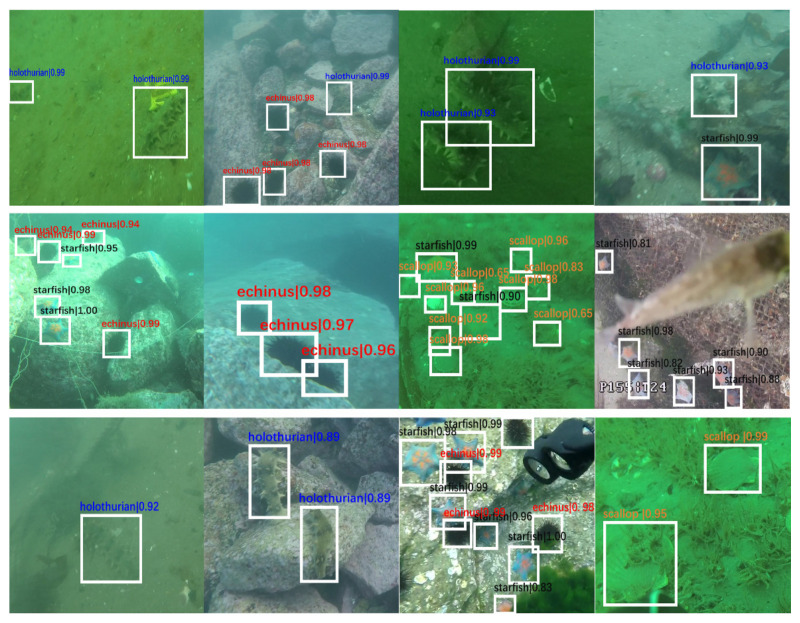
Test results for the holothurian, echinus, scallop, and starfish.

After completing the pre-training of each sub-module, the overall framework is applied to the test set of the underwater data set. The partial measured results obtained are shown in Figure 7.

A dynamic threshold two-stage network is used in the detection part in the proposed framework, and the detection results of the four types of marine organisms in the data set are closely related to the selection of the IoU threshold. Thus, the IoU threshold is constantly changed in the course of the experiment so as to ascertain the detection precisions under different IoU thresholds (Table 1 and Table 2). 

Table 1 shows the detection results when the IoU threshold is fixed at 0.5. Table 1 indicates that for different underwater environments, the detection recall rates of the four marine organisms are 0.759, 0.900, 0.735, and 0.827. Therefore, the proposed framework can detect and recognize most underwater targets to be detected. Table 2 shows the mean average precision among different types of marine organisms when the IoU threshold varies. When the IoU threshold is 0.5, the mAP of the entire model is 0.694, thereby indicating that the detection precision of the proposed framework is high, and the probability that the target can be correctly detected is also very high. Furthermore, when the threshold of IoU is 0.7, mAP can still reach 0.538, and the mAP@ [0.5:0.05:0.95] of the entire underwater test set can reach 0.408.

### 5.2. Ablation Experiment

To further verify the effectiveness of the UIEM, ablation experiments are also conducted. The assistance of each sub-module in the UIEM to the final detection result is shown by adding different sub-modules. Six groups of ablation experiments are conducted, and the experimental results are shown in Table 3. 

**Figure 8:**
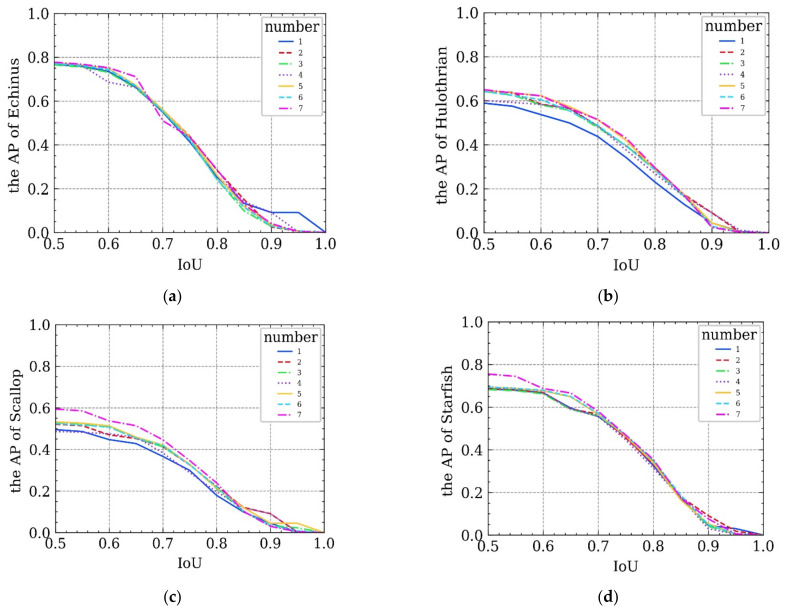
Ablation experiments results: (**a**–**d**) corresponds to the trend of the detection precision of each organism category in the data set changed with the IoU threshold during the ablation experiments in six groups.

During the ablation experiments for the six groups, the trend of the detection precision of each category in the data set changed with the IoU threshold (Figure 8). The ablation experiments confirm that any one of the three sub-modules for denoising, deblurring, and color correction is helpful for the detection performance of the overall framework. The highest detection precision of the overall framework is reached when all three sub-modules are added. Therefore, each of the three sub-modules has its own role. After being added to the overall framework, they complement one another and significantly improve the performance of the proposed framework for underwater target detection.

### 5.3. Comparative Test

**Figure 9:**
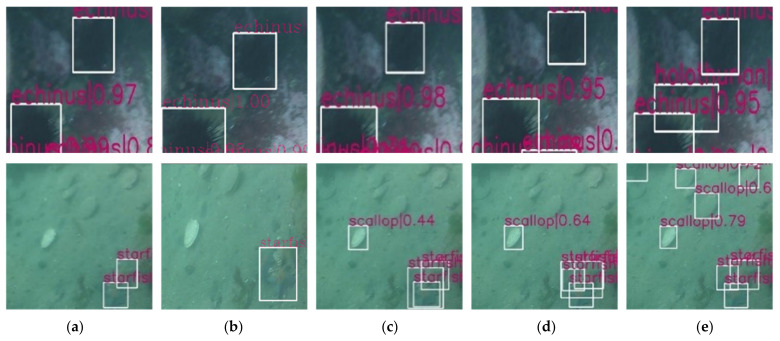
Comparison results of different model detections: (**a**) SSD, (**b**) Yolo_v3, (**c**) Faster RCNN, (**d**) Cascade RCNN (**e**) proposed framework.

In the comparative experiment, the proposed framework is evaluated against models that have been practically applied in marine organism detection as regards detection precision and detection speed under the same data set, including SSD [41], YOLO_v3 [42], Faster RCNN [43], and Cascade RCNN [44]. In the comparative experiment, SSD uses VGG16 as the backbone, Yolo_v3 uses darknet53 as the backbone, and Faster RCNN and Cascade RCNN both use resnet50 as the backbone. For model training, we use SGD optimizer with learning_rate = 0.01, momentum = 0.9 and weight_decay = 0.0001. The comparative experimental results are shown in Figure 9 and Table 4, Table 5 and Table 6.

Figure 9 is a sample diagram of the detection results of different networks for the same data set. Given the severe image degradation, the distinction between the foreground and background is poor. Other models perform poor detection and recognition effects for scallops partially buried in sand and holothurians with similar background colors. Moreover, as the UIEM is introduced in the proposed framework, these severely degraded images are enhanced, so that the ability of target detection in these images is greatly enhanced, thereby improving detection precision.

Table 4 and Table 5 show the comparison of the detection precisions and recalls of different networks for various marine organism in the same data set. As all networks are in accordance with region proposals, the detection precision will be varied under different IoU thresholds. Therefore, in Table 4, a comparison of detection precision under two IoU thresholds is respectively given. It can be seen from Table 4 that the mAP@ [0.5:0.05:0.95] of the proposed framework has reached 0.408, which is a significant improvement compared to other detection networks that have been applied to marine organism detection. Meanwhile, due to the introduction of the UIEM, the mAP of the proposed framework can still reach 0.538 even when the threshold of IoU is 0.7, which is 6 percentage higher than the Cascade RCNN. The effectiveness of the UIEM is further verified.

In addition, to compare the detection speeds of different networks, the operating frame rate of each network in the same experimental platform is tested. The test results are shown in Table 6. The proposed framework can still meet the needs of real-time detection even with the inclusion of the UIEM.

## 6. Conclusions and Future Work

This study proposes an end-to-end marine organism detection framework that can jointly optimize the image enhancement and target detection. With a two-stage target detection network as the backbone, an underwater image enhancement module consisting of denoising, color correction, and deblurring sub-modules are added into the framework, with an expectation of considerably improving the ability of the framework to deal with severely degraded underwater images caused by poor underwater lighting conditions. Meanwhile, the pre-training of the underwater image enhancement module is completed with the help of self-built data sets so that end-to-end training of the entire framework can be performed, and the joint optimization of the enhancement module and the subsequent target detection module can be achieved. Given the difficulty of detection caused by the uneven distribution of sizes and positions of different types of marine organisms in underwater images, the feature pyramid network composed of multiple ResNets is introduced in the target detection part of the proposed framework. High-quality feature extraction of marine organisms can be achieved by combining deep semantic information and shallow detail information at different levels. To enable the proposal region in the two-stage network of the detection module to better match marine organisms of different scales, dynamic label allocation and dynamic smoothing of L1 loss are introduced to achieve dynamic adjustment of the IoU threshold, thereby greatly improving target detection and model training precision. Compared with the existing end-to-end models applied to marine organism detection, the framework proposed in this study can increase the detection precision by at least 6% because of the introduction of the image enhancement network. Meanwhile, the detection speed of the proposed framework dropped slightly, taking into account both the detection performance and real-time performance, so that the framework is applicable to the underwater mobile visual observation platforms for high-precision real-time detection of marine organisms.

In order to improve the proposed framework’s ability to process images under harsh underwater conditions, we have added UIEM composed of three sub-modules. To balance the detection speed and the detection performance, we have to use a relatively simple network structure to construct the three sub-modules in UIEM, which makes the enhancement effect unsatisfactory. Therefore, future work will mainly focus on how to use a single network structure with better performance to build UIEM to achieve better underwater image enhancement effects, so as to greatly improve the performance of marine organism detection under the premise of real-time detection.

## Figures and Tables

**Table 1:** Recall and average precision of the underwater data set with an IoU threshold of 0.5 for the proposed framework.

Class	Ground Truths	Detections	Recall	AP
Holothurian	626	1849	0.759	0.650
Echinus	3228	9997	0.900	0.777
Scallop	4206	8576	0.735	0.594
Starfish	1834	3367	0.827	0.755

**Table 2:** Mean average precision of the underwater data set at different IoU thresholds of the proposed framework.

	IoU	0.50	0.55	0.60	0.65	0.70	0.75	0.80	mAP@ [0.5:0.05:0.95]
Class									
Holothurian		0.650	0.635	0.622	0.566	0.514	0.429	0.296	0.408
Echinus		0.777	0.768	0.752	0.711	0.510	0.435	0.287	
Scallop		0.594	0.585	0.537	0.514	0.446	0.346	0.239	
Starfish		0.755	0.744	0.687	0.666	0.580	0.469	0.355	
mAP		0.694	0.683	0.650	0.614	0.538	0.420	0.295	

**Table 3:** Ablation experiment results of the three sub-modules. The “√” indicates that the corresponding sub-module has been added to the framework.

	Denoising	Deblurring	Color Correction	mAP@0.5	mAP@0.7
①				0.634	0.477
②	√			0.656	0.506
③		√		0.655	0.501
④			√	0.638	0.495
⑤	√	√		0.662	0.515
⑥		√	√	0.657	0.507
⑦	√	√	√	0.694	0.538

**Table 4:** Detection precision of different models for various underwater targets with IoU thresholds of 0.5 and 0.7.

	IoU = 0.5				IoU = 0.7						mAP@ [0.5,0.95]
	Holothurian	Echinus	Scallop	Starfish	mAP	Holothurian	Echinus	Scallop	Starfish	mAP	
**SSD**	0.487	0.682	0.421	0.676	0.567	0.352	0.442	0.253	0.462	0.377	0.309
**Yolo_v3**	0.577	0.691	0.442	0.599	0.577	0.368	0.442	0.297	0.392	0.375	0.305
**Faster RCNN**	0.587	0.767	0.489	0.688	0.633	0.433	0.543	0.358	0.526	0.465	0.358
**Cascade RCNN**	0.589	0.767	0.495	0.685	0.634	0.437	0.549	0.366	0.556	0.477	0.373
**Proposed framework**	0.650	0.777	0.595	0.755	0.694	0.514	0.610	0.446	0.580	0.538	0.408

**Table 5:** Detection recall of different models for various underwater targets with IoU thresholds of 0.5 and 0.7.

	IoU = 0.5				IoU = 0.7			
	Holothurian	Echinus	Scallop	Starfish	Holothurian	Echinus	Scallop	Starfish
**SSD**	0.740	0.889	0.702	0.808	0.490	0.614	0.401	0.568
**Yolo_v3**	0.577	0.691	0.442	0.599	0.368	0.442	0.297	0.392
**Faster RCNN**	0.716	0. 859	0.621	0.774	0.564	0.681	0.483	0.619
**Cascade RCNN**	0.728	0.847	0.633	0.785	0.567	0.681	0.499	0.637
**Proposed framework**	0.759	0.900	0.735	0.827	0.604	0.722	0.588	0.690

**Table 6:** Detection frame rate of different networks.

Model	SSD	Yolo_v3	Faster RCNN	Cascade RCNN	Proposed Framework
**Fps**	57	70	43	38	41

## Data Availability

Not applicable.
